# Comprehensive transcriptome analyses reveal tomato plant responses to tobacco rattle virus-based gene silencing vectors

**DOI:** 10.1038/s41598-017-10143-1

**Published:** 2017-08-29

**Authors:** Yi Zheng, Biao Ding, Zhangjun Fei, Ying Wang

**Affiliations:** 1 Boyce Thompson Institute, Cornell University, Ithaca, NY 14853 USA; 2 Department of Molecular Genetics, The Ohio State University, Columbus, OH 43210 USA; 3 USDA-ARS Robert W. Holley Center for Agriculture and Health, Ithaca, NY 14853 USA; 4 Department of Biological Sciences, Mississippi State University, Starkville, MS 39759 USA

## Abstract

In plants, virus-induced gene silencing (VIGS) is a popular tool for functional genomic studies or rapidly assessing individual gene functions. However, molecular details regarding plant responses to viral vectors remain elusive, which may complicate experimental designs and data interpretation. To this end, we documented whole transcriptome changes of tomato elicited by the application of the most widely used tobacco rattle virus (TRV)-based vectors, using comprehensive genome-wide analyses. Our data illustrated multiple biological processes with functional implications, including (1) the enhanced activity of miR167 in guiding the cleavage of an auxin response factor; (2) reduced accumulation of phased secondary small interfering RNAs from two genomic loci; (3) altered expression of ~500 protein-coding transcripts; and (4) twenty long noncoding RNAs specifically responsive to TRV vectors. Importantly, we unraveled large-scale changes in mRNA alternative splicing patterns. These observations will facilitate future application of VIGS vectors for functional studies benefiting the plant research community and help deepen the understanding of plant-virus interactions.

## Introduction

Molecular tools that modulate gene expression are critical for biological research and applications. Albeit various tools are available for such purposes in plant biology, virus-induced gene silencing (VIGS) has been widely used thanks to its effectiveness and ease of handling^1–5^. The principle of VIGS rests on the phenomenon that plant RNA silencing machinery cleaves viral RNA to a population of virus-derived small RNAs (vsRNAs)^6^. The vsRNAs are integrated into host gene silencing effectors (Argonaute proteins) for function. When a plant gene fragment is inserted into viral sequences, vsRNAs generated from the inserted sequences will guide the cleavage of host transcripts based on sequence homology, leading to decreased accumulation of target genes^7^. Tobacco rattle virus (TRV)-based VIGS vectors were among the first that have been developed, and have been widely applied to functional gene studies in various plants^8–12^. The TRV vectors (pTRV1 and pTRV2), by removal of non-structural coding genes in the RNA2 genome, maintain the infectivity and facilitate the insertion of plant gene fragments^13^. When using TRV vectors, a typical design includes using agrobacteria harboring pTRV1 plus either empty pTRV2 vector or a pTRV2-GFPsil as controls to compare phenotypes obtained from treatments with TRV-target constructs. While this experimental setup is generally acceptable to many purposes, it remains largely elusive regarding the plant responses to the presence of TRV or TRV-based vectors at the whole transcriptome level. A recent report using microarrays showed that TRV infection activated the hypersensitive response pathway in potato tubers, which demonstrated the alteration of host protein-coding genes by TRV but did not examine the dynamics of other important regulatory noncoding RNAs such as small RNAs and long noncoding RNAs (lncRNAs)^14^.

Virus infection generally affects the accumulation of certain plant miRNAs, depending on the host and virus combinations. For example, miR168 is ubiquitously up-regulated in most of plant-virus combinations with a few exceptions while miR158 can be only elicited by turnip mosaic virus but not tobacco mosaic virus or cucumber mosaic virus (collectively reviewed in Yin *et al*.^15^). However, whether the guided cleavage of plant miRNAs is affected by viruses or not has not been well studied at the transcriptome level. In addition, little is known regarding the genome-wide expression patterns of lncRNAs in various host-virus combinations except those in plants infected with a nuclear-replicating viroid^16^ or a DNA virus^17^. Furthermore, most, if not all, of VIGS vectors are composed of engineered viral sequences which may trigger distinct plant responses as compared with those triggered by wild type (WT) viruses. Therefore, we reason that a comprehensive understanding of plant responses to TRV vectors is in need to facilitate future experimental designs.

To this end, we used next-generation sequencing technologies to outline comprehensive transcriptome profiles (small RNAs, mRNAs, and degradome RNAs) in tomato plants applied with TRV vectors. Thus, this study is valuable for future experimental designs benefiting the plant research community and provides new insights into plant-virus interactions.

## Results and Discussion

### Comprehensive transcriptome sequencing captures tomato responses to the application of TRV vectors

**Figure 1:**
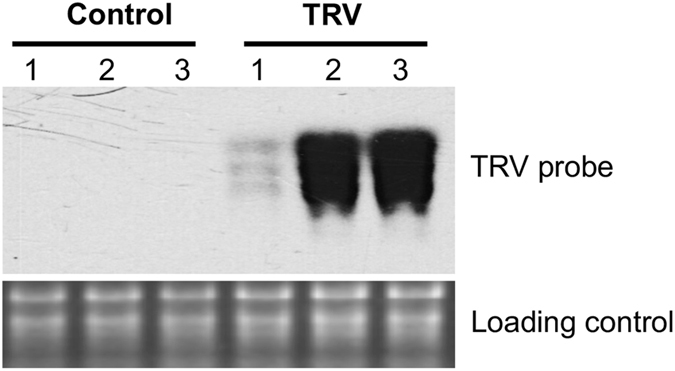
Northern blots showing the presence of TRV in tomato leaf samples after 3 weeks post inoculation. Three biological replicates for mock and TRV vectors-inoculated samples using individual plants were presented.

We inoculated tomato seedlings with a mixture of agrobacteria harboring equal amount of pTRV1 and pTRV2-GFPsil vectors and verified the presence of TRV in the top leaves at three weeks post inoculation (Fig. 1). We used a mixture of top three leaves from each individual plant as a biological replicate and three biological replicates each for mock and TRV vectors-inoculated samples. As an advancement, we used RNA from the same samples to generate three sets of libraries for sequencing: sRNA, degradome RNA (dRNA), and RNA-Seq, which allowed us to analyze the data in a coordinated manner for unraveling the expression and function of various RNA populations. After processing the sequencing data, for each sample we obtained ~1–3 million high-quality sRNA reads, 2.5–13 million dRNA-Seq reads, and 4.4–15.7 million RNA-Seq reads (Supplementary Table S1). These data together captured the dynamics of various classes of regulatory RNAs and protein-coding mRNAs.

### TRV vectors impact the expression and function of tomato sRNAs

**Figure 2:**
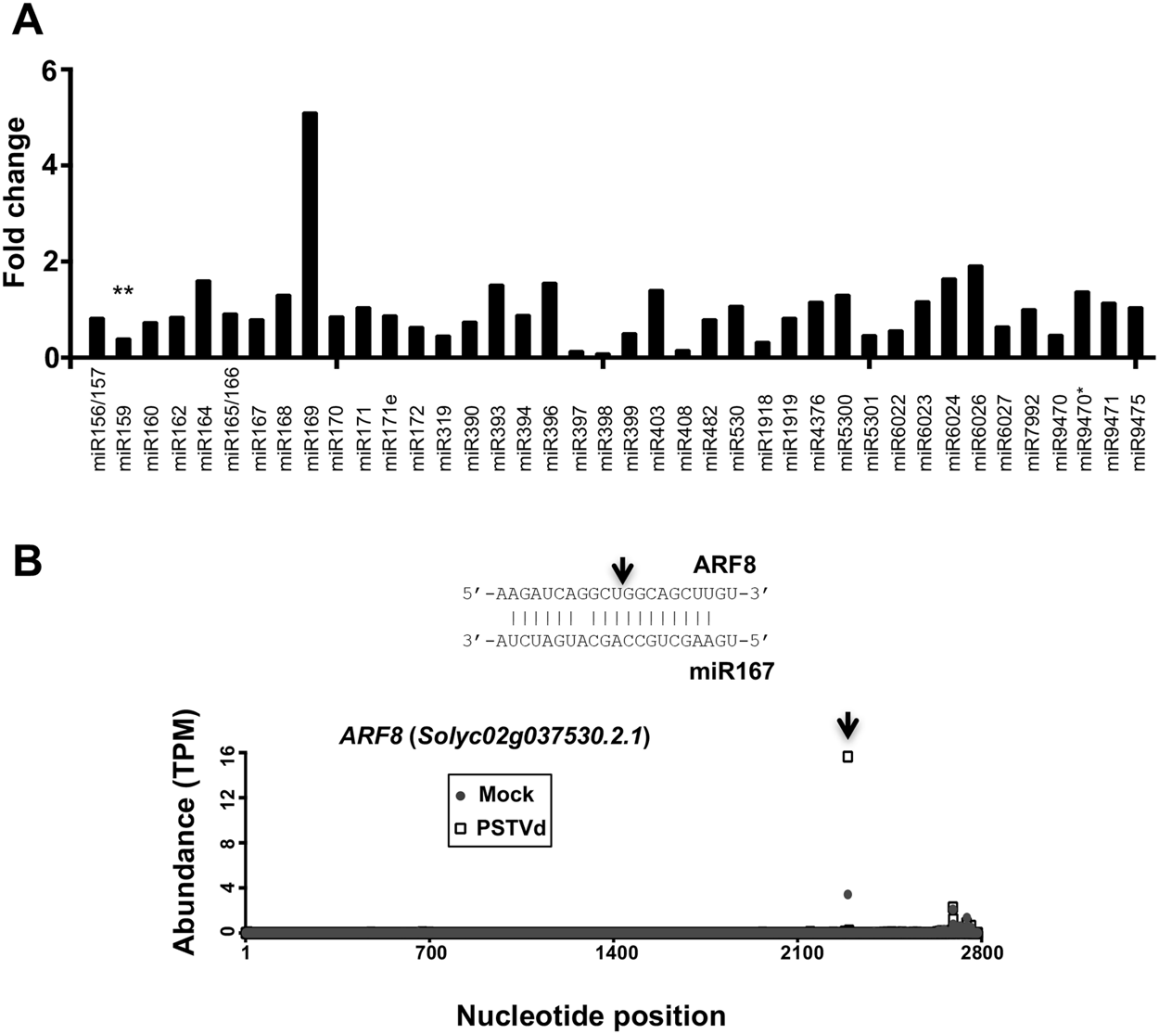
Expression and cleavage activity dynamics of tomato miRNAs upon TRV inoculation.(**A**) MiRNA expression changes in TRV vectors-inoculated samples compared with mock samples. **Indicates P < 0.01.(**B**) Change in miR167-guided cleavage activities detected with PARE data. MiR167 and target pairings are shown in the top panel. Arrows indicate the guided cleavage positions. Abundances of degradome tags are shown in the bottom panel.

sRNAs, including microRNAs (miRNAs) and small interfering RNAs (siRNAs), are essential regulators involved in various biological processes. Extensive studies revealed that viral infection affects the expression of certain plant miRNAs^15^. We tested whether TRV vectors also affect the expression and function of plant sRNAs. We found that only miR159 among all known tomato miRNAs showed a significantly decreased expression upon TRV inoculation based on the normalized read numbers from three biological replicates (Fig. 2a; Supplementary Table S2). This pattern is in contrast to the induction of tomato miR171e and miR4376 in response to the infection of PSTVd^16^, attesting to the specific host responses. Interestingly, miR159 appears to be a common target by various plant viruses but exhibits diverse expression patterns in different host-virus combinations^15^. Furthermore, miR159 can directly interact with a silencing suppresser encoded by African cassava mosaic virus when infecting Arabidopsis^18^, which suggests that some viruses can compromise the function of miR159 without changing its accumulation level. Besides miRNAs, we are also interested in phasiRNAs, which are a unique class of plant siRNAs derived from truncated transcripts as products of miRNA-guided cleavages and arranged in a head-to-tail phased pattern^19, 20^. The phasiRNA pathway is implicated in regulating plant innate immunity via regulating various NBS-LRR family genes^21–23^. We identified 86 phasiRNA generating loci (PHAS) and uncovered miRNA/sRNA triggers for 28 of them (Supplementary Table S3). None of the trigger miRNAs/siRNAs showed significant changes in their accumulation levels in response to TRV vector inoculation. However, we found that the abundance of phasiRNAs generated from two PHAS loci showed 2-fold changes with P values below 0.05 (Supplementary Table S3). One PHAS locus mapped to a calcium-dependent protein kinase (*Solyc01g096350*) and the other mapped to a receptor-like kinase (*Solyc12g100010*), both of which are likely involved in plant defense signaling. Noteworthy is that, in contrast to the expression changes in seventeen PHAS loci upon PSTVd infection, TRV vectors have a milder impact on the expression of tomato phasiRNAs. Interestingly, the abundance of phasiRNAs generated from the PHAS locus mapped to a receptor-like kinase (Solyc12g100010) was also reduced in PSTVd-infected tomato plants^16^, therefore this PHAS locus might be a common target for infectious RNAs involved in host-virus/viroid interactions.

To evaluate the function of host sRNAs in response to TRV vectors, we performed parallel analysis of RNA ends (PARE) of our dRNA data to uncover sRNA-guided cleavages of mRNAs. Statistical analysis by the CleaveLand Suite^24^ identifies sRNA-guided transcript cleavages and provides a calculated p-value as well as a category score ranking from 0 (most promising) to 5 (least promising). In general, categories 0 and 1 can be considered as a strong support of sRNA-guided cleavage. Using our deep sequencing data of sRNAs (21- and 22-nt populations) and dRNAs, we uncovered 798 positive events of sRNA-guided transcript cleavages satisfying both the P value < 0.05 in at least one sample and a category score of 1 or 0 in at least one sample (Supplementary Table S4).

In general miRNA activities remained largely unaffected when examining the category scores as well as the accumulation of the cognate target transcripts (Supplementary Table S5). For example, miR396 regulation over *Solyc12g096070* (encoding GROWTH-REGULATING FACTOR 5/GRF5) had a category score of 0 in all mock and TRV vectors-inoculated samples and had similar accumulation levels of the target *GRF5* transcripts in all samples. These serve as positive controls supporting the quality of the data. Despite the reduction of miR159, the miR159-guided cleavage was slightly hampered for transcripts encoding an unknown protein (*Solyc12g014120*) but unaffected for transcripts encoding a GAMYB-like protein (*Solyc06g073640*), which may be attributed to the high abundance of miR159 even with repression triggered by TRV vectors. Interestingly, we found that the activities were significantly enhanced for miR167-guided cleavage of transcripts encoding tomato auxin response factor 8/ARF8 (*Solyc02g037530*) when inoculated with TRV vectors. While all TRV vectors-inoculated samples had category 0 for this regulation, two samples had category 2 and one sample had category 0 in mock. This enhanced activity was significant (P < 0.01) and resulted in a 40% reduction in the *ARF8* accumulation (Fig. 2b; Supplementary Table S5). In the meantime, the activity of miR167 in regulating other genes was not affected. Interestingly, miR167 is a common target for a broad spectrum of plant pathogens including a viroid^16^, viruses^25, 26^, bacteria^27^, and fungi^28^. Thus, our data, together with the previous observations, further consolidate the ubiquitous role of miR167-based regulation over *ARF8* in plant-pathogen interactions.

### TRV vectors globally affect the expression and alternative splicing patterns of protein-coding transcripts

Viral infection often triggers the global expression changes of genes involved in stress response, cell wall structure, chloroplast function, protein metabolism, and hormonal pathways^16, 29–32^. In this study, we employed RNA-Seq analysis to achieve a high resolution for dynamic changes in gene expression profiles in response to TRV vectors in the tomato cultivar Heinz 1706. We identified 175 and 313 genes that were significantly up- or down-regulated in response to TRV vectors, respectively (Supplementary Table S6). The number of affected genes in our study is comparable to 439 probes/genes affected in WT TRV-infected potato tubers using microarray analysis^14^. Pathway analysis found that anthocyanin metabolism, and formate metabolism were repressed while energy metabolism (such as glycolysis and photosynthesis) was induced (Supplementary Table S7). Interestingly, we did not observe the global activation of the hypersensitive response pathway in TRV vectors-inoculated as was reported for WT TRV infection of potato tubers^14^. This difference is likely attributable to the deletion of viral sequences in TRV vectors as well as distinct host-virus combinations.

**Figure 3:**
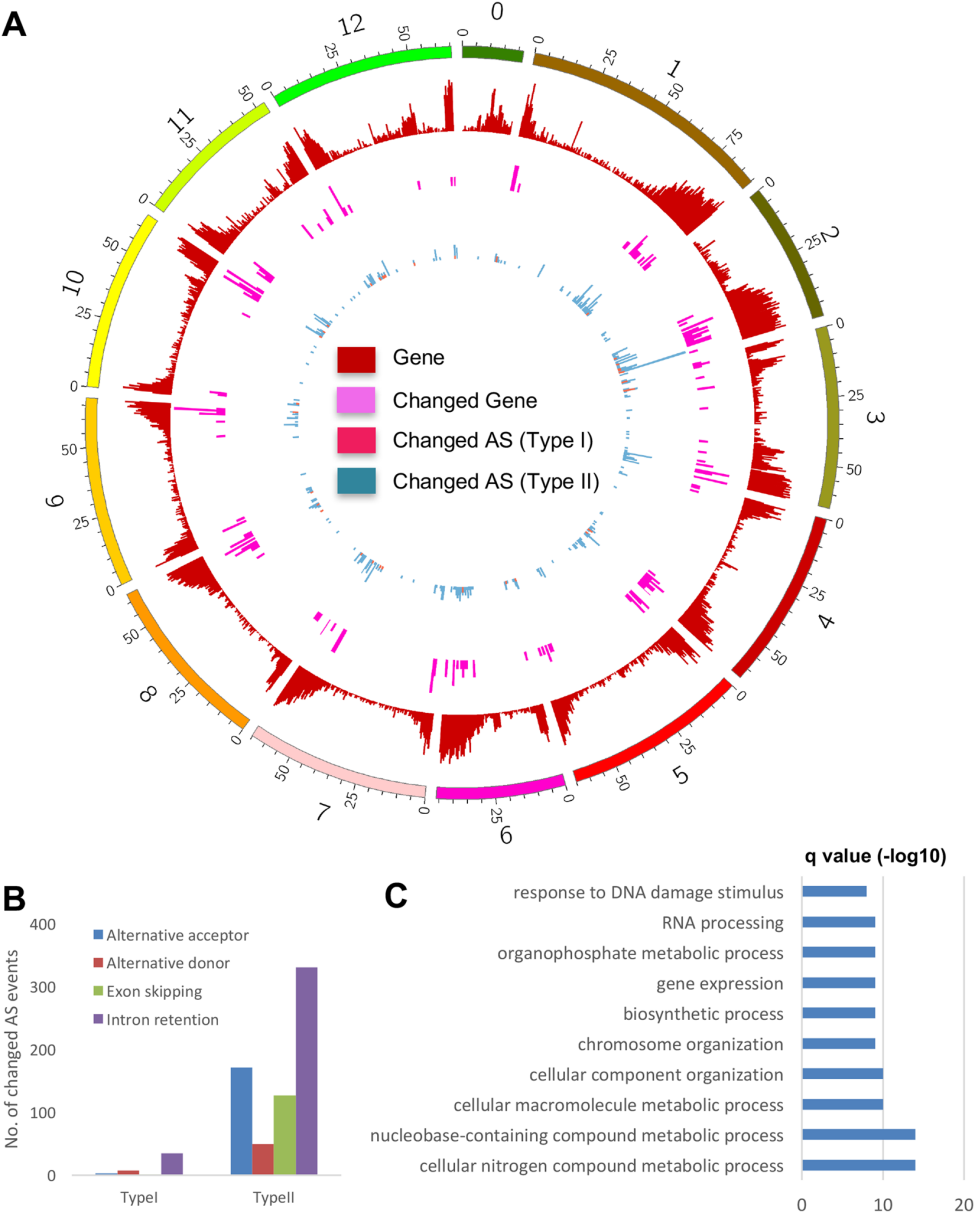
Gene expression dynamics and large-scale changes in alternative splicing (AS) upon TRV inoculation.(**A**) Density plot of protein-coding genes, differentially expressed protein-coding genes, and protein-coding genes with changed AS (Type I and Type II) across the tomato genome.(**B**) Summary of different categories of changed AS events.(**C**) Top 10 GO terms significantly enriched in protein-coding genes with changed AS.

Noteworthy is that we found the differential expression of some protein-coding genes in association with changes in alternative splicing (AS) events. Two types of AS changes were observed: 1) splicing patterns were the same between mock and infected samples but only one of the splicing variants showing significant changes in expression; and 2) splicing patterns changed directly between mock and infected samples. We found that 36 loci have only one splicing variant selectively up- and down-regulated (Type I changes) (Fig. 3a; Supplementary Table S8). The Type II changes included exon skipping, alternative 5′ donor sites, alternative 3' acceptor sites, and intron retentions. We identified 347 loci that showed distinct alternative splicing patterns between mock and inoculated samples (Fig. 3a; Supplementary Table S9), among which intron retention was the most dominant AS events being affected, while exon skipping and alternative acceptor each accounted for one-fourth of the AS events being affected (Fig. 3b). Gene ontology analysis showed that the genes with AS changes (Types I and II) were predominantly involved in metabolic processes and control of gene expression at multiple levels (Fig. 3c; Supplementary Table S10), indicating that inoculation of TRV vectors affects cellular processes through altering both the primary sequences and expression of regulatory and metabolic gene products. AS is a pivotal layer of gene regulation to attenuate compositions of various cellular machinery for functions, therefore virus affecting AS patterns represents a new mechanism underlying the molecular dynamics in plant-virus interactions. Recent studies showed that PSTVd^16^ as well as wildtype panicum mosaic virus together with its satellite virus^33^ can induce global changes of AS in host transcriptome affecting the expression of hundreds of genes. Here we found that TRV vectors, after the deletion of several non-structural genes, retained the capacity to change large-scale AS patterns in tomato transcriptome, showing that changes in AS patterns likely represent a common mechanism in altering host transcriptome. This information is also valuable for future experimental designs, when using TRV VIGS vectors, to consider the AS changes when assessing the expression of target genes (e.g. facilitate the primer designs for qRT-PCR assays, etc).

### LncRNAs show specific expression patterns in response to TRV vectors

Accumulative evidence begins to unravel the pivotal role of lncRNAs in important biological processes, and TRV vectors-based VIGS can serve as a powerful tool to rapidly analyze the function of lncRNAs in plants. However, the impact of viral infection on the expression of plant lncRNAs remains unclear for most of the plant-virus combinations and the knowledge just started to emerge. Recent studies using RNA-Seq approach documented the genome-wide expression patterns of tomato lncRNAs in response to a DNA virus^17^ or a viroid^16^. In terms of plant response to RNA viruses, turnip crinkle virus infection affects the coupled expression pattern of a lncRNA and the nearby APETALA2 gene in Arabidopsis, but it is unclear for the global lncRNA expression changes at the transcriptome level in the report^34^. Using the RNA-Seq data generated in this study, we identified 6,894 genomic loci that generated detectable lncRNAs (Supplementary Table S11), the majority of which were identical to the ones reported in a recent study^16^. We then focused on those that were specifically responsive to TRV vectors using a stringent criterion (see methods for details), and found twenty such transcripts (Supplementary Table S12). A comparison of PSTVd-responsive and TRV-responsive lncRNAs in tomato showed that six lncRNAs are commonly suppressed upon infection, implying shared genomic regions subject to transcriptional repression in response to infectious RNAs. Our recent work showed that the expression patterns of seven lncRNAs were tightly coupled with their adjacent protein-coding genes in PSTVd-infected leaves^16^. Such phenomenon implies *trans*-regulation of these lncRNAs to their adjacent genes. However, we did not observe any of such examples in TRV vectors-inoculated samples.

In conclusion, we found that TRV vectors elicit the changes in the function of miR167, the abundance of phasiRNAs from two PHAS loci, expression patterns of over hundred protein-coding RNAs and twenty lncRNAs, as well as changes in alternative splicing patterns of 383 host transcripts. The information will benefit the plant research community in future experimental designs when using TRV VIGS vectors. The data also shed lights on the new layers of gene regulation in plant-virus interactions by providing genome-wide information in altered AS events and specific lncRNAs responsive to TRV derivatives.

## Methods

### Plant materials

Tomato plants (cv. Heinz 1706) were grown in a greenhouse at 25 °C and with a 16/8 hr light/dark cycle. Seedlings with first two true leaves just emerging were inoculated with water or agrobacterium containing pTRV1 (TAIR accession number CD3–1039) and pTRV2-GFPsil (TAIR accession number CD3–1044). After three weeks post-inoculation, top three leaves from each plant were collected and the TRV infection was verified by Northern blotting. There were three biological replicates for mock samples and three for TRV vectors-applied samples as well.

### Total RNA isolation and enrichment for sRNAs

Total RNA from collected leaf samples was isolated and fractioned to >200 nt and <200 nt populations using the RNAzol^RT^ reagent (Sigma-Aldrich, St. Louis, MO). sRNA species were further purified using the mirVana miRNA isolation kit (Thermo Fishier Scientific, Grand Island, NY) following the manufacturer’s instructions. mRNA populations were further purified using the Magnetic mRNA isolation kit (NEB, Ipswich, MA).

### Northern blots

Total RNA was run on 1% (w/v) denaturing agarose gels, transferred to Hybond-XL nylon membranes using a vacuum blotting system (Amersham Biosciences, Little Chalfont, UK), and then immobilized by UV cross-linking. After blocking and overnight hybridization to [a-^32^P] UTP-labeled riboprobes at 60 °C in ULTRAhyb reagent (Thermo Fishier Scientific, Grand Island, NY), the membranes were washed four times and exposed to a storage phosphor screen (Kodak, Rochester, NY). For detecting TRV, probes were obtained by transcribing *in vitro* NotI-linearized pCR4-TRV1^6732–6791^ template using a T3 MAXIscript kit (Thermo Fishier Scientific, Grand Island, NY).

### Library construction and sequencing

sRNA libraries were constructed following the established protocol^35^. Briefly, 18–30 nt sRNA populations were purified using 17% urea-PAGE gel and then subject to ligation with 3′- and 5′-adapters. sRNA populations with adapters were reverse transcribed, PCR amplified, and then purified from 8% native-PAGE gels. dRNA libraries were generated following the established protocol with minor modifications^24^. Briefly, a 5′-RNA adapter (5′-cagaguucuacaguccgacgauccagcag-3′) containing an Ecop15I site was ligated to the 5′-end of sRNA-guided cleaved mRNA species (dRNAs). An oligo d(T) primer (5′-ctgatctagaggtaccggatcccagcagt-3′) containing another Ecop15I site was used for reverse transcribing the dRNAs followed by PCR amplification. The amplified products were digested with Ecop15I (New England Biolabs, Ipswich, MA), followed by urea-PAGE gel purification of 97-mers, 3′-DNA adapter ligation, and PCR amplification. Strand-specific RNA-Seq libraries were constructed using the protocol described previously^36^. All the library constructs were analyzed and quantified by bioanalyzer and sequenced on an Illumina HiSeq. 2500 system.

### RNA-Seq read processing, transcript assembly, and differential expression

Paired-end RNA-Seq reads were processed to remove adapters, low-quality bases using Trimmomatic^37^, and reads shorter than 40 bp were discarded. The remaining high-quality reads were subject to rRNA sequence removal by aligning to an rRNA database^38^ using Bowtie^39^ allowing up to three mismatches. The resulting read pairs were aligned to the tomato genome using Tophat2^40^ allowing up to two mismatches. Only aligned read pairs with no mismatch were used to assemble into transcripts using Cufflinks^41^. The expression of transcripts was calculated and normalized to FPKM (fragments per kilobase of exon per million mapped fragments) base on all mapped read pairs using Cuffnorm, a component in the Cufflinks package. Differential expression analysis was conducted using Cuffdiff, a component in the Cufflinks package. Protein-coding genes with adjusted P < 0.05 and fold changes of at least 2 were considered differentially expressed.

### Functional annotation and coding potential assessing of assembled transcripts

The assembled transcripts were BLAST against the *Arabidopsis thaliana* protein^42^ and the UniProt (TrEMBL and SwissProt) databases^43^ with an E-value cutoff of 1e-4. The Coding Potential Calculator (CPC)^44^ was used to assess coding potential of the transcripts.

### Identification of lncRNAs and alternative splicing events

Assembled transcripts derived from the tomato gene models or shorter than 200 bp were excluded from this analysis. The remaining transcripts were digitally translated into proteins in 3 forward frames, and the longest amino acid sequences were used to determine the ORF length. We define lncRNAs by satisfying both CPC scores <0 and ORF lengths <100. Differentially expressed lncRNAs were identified using the same approach for protein-coding genes as described above except that we required lncRNAs to be induced or repressed in all three replicated samples due to their stochastic expression.

Alternative splicing (AS) events were identified from the expressed isoforms using ASTALAVISTA^45^. Different types of AS events were extracted and counted as previously described^46^. Briefly, we searched for four categories of AS events, included exon skipping, alternative 5′ donor sites, alternative 3′ acceptor sites, and intron retentions. We analyzed the abundance changes of alternatively spliced transcripts from the same locus as well as AS pattern changes from the same locus at a genome-wide scale.

### sRNA sequence processing

sRNA reads were processed to remove adaptors, low-quality bases, and short reads (less than 15 nt). The resulting sRNA reads were further filtered by removing those mapped to the sequences of tRNAs, snoRNAs, snRNAs (collected from GenBank) or rRNAs^38^ using Bowtie^39^. Raw counts for each unique sRNAs were derived and normalized into TPM (transcripts per million). sRNAs that were expressed at >5 TPM in at least one sample were processed using DESeq.^47^ to identify differentially expressed sRNAs between mock and TRV vectors-inoculated tomatoes. sRNAs satisfying both adjusted p values <0.05 and fold changes > = 2 were considered as differentially expressed.

### Identification of miRNAs

We followed a previously described method^48^ to identify miRNAs from cleaned sRNA reads. sRNAs with >10 TPM in at least one sample were mapped to the tomato genome (ver. SL2.40) using Bowtie^39^ allowing no mismatch. We discarded sRNAs mapped to more than 20 loci in the genome. The mapped loci and 200 bp flanking sequences on each side were extracted and then folded *in silico* using RNAfold^49^. Resulting folded structures were checked with miRcheck^50^ to uncover candidate miRNAs, which were further compared with miRBase^51^ to identify conserved miRNAs.

### Identification of candidate PHAS loci

The method we used was previously described in details^19^. In brief, the cleaned sRNA sequences were mapped to the tomato reference sequences (genome or transcriptome) using Bowtie^39^ allowing no mismatch and no more than six hits. The reference sequences were then scanned with a sliding window of 189 bp (nine 21-nt phase registers). A positive PHAS window was identified if it contains no less than 10 unique sRNAs, with more than half of the unique sRNAs being 21-nt in length and with no less than three 21-nt unique sRNAs falling into the phase registers. We combined those windows when they shared the same phase registers and fell into the same gene loci. P values and phasing scores for positive windows were calculated following the methods described previously^52, 53^.

### dRNA read processing and identification of cleavage sites

dRNA data were processed to filter out adapters, low-quality bases, short reads (<15 nt), as well as those mapped to the rRNA database. We aligned the cleaned dRNA reads to the assembled transcripts in order to generate degradome density file. We also identified the cleavage sites of miRNAs and siRNAs using CleaveLand Suite v4.3^24^.

### Accession numbers for deep sequencing data

The raw sequences of sRNAs, dRNA and RNA-Seq have been deposited in the NCBI SRA with the accession numbers SRP093503 (control samples) and SRP103592 (TRV vectors-inoculated samples).

## Electronic supplementary material

Dataset 1

Dataset 2
