# Supplementary material for: Comprehensive transcriptome analyses reveal tomato plant responses to tobacco rattle virus-based gene silencing vectors
Source: Sci Rep. 2017 Aug 29;7:9771. doi: 10.1038/s41598-017-10143-1 (PMC5575331; doi:10.1038/s41598-017-10143-1)
Supplement: Supplementary file 2 — Dataset 2 [file 41598_2017_10143_MOESM2_ESM.pdf]

Comprehensive transcriptome analyses reveal tomato plant responses to tobacco rattle virus-based gene silencing vectors

Yi Zheng,[1,5] Biao Ding, [2,6] Zhangjun Fei,[1,3,*] Ying Wang[2,4,5,*]

[1] Boyce Thompson Institute, Cornell University, Ithaca, NY 14853, USA
[2] Department of Molecular Genetics, The Ohio State University, Columbus, OH 43210, USA
[3] USDA-ARS Robert W. Holley Center for Agriculture and Health, Ithaca, NY 14853, USA
[4] Department of Biological Sciences, Mississippi State University, Starkville, MS 39759, USA
[5] These authors contributed equally to this work.
[6] Deceased.

[*] Correspondence should be addressed to Zhangjun Fei (zf25@cornell.edu) or Ying Wang (wang@biology.msstate.edu).

Supplementary Data Set 1

Figure 1

Probe against TRV1

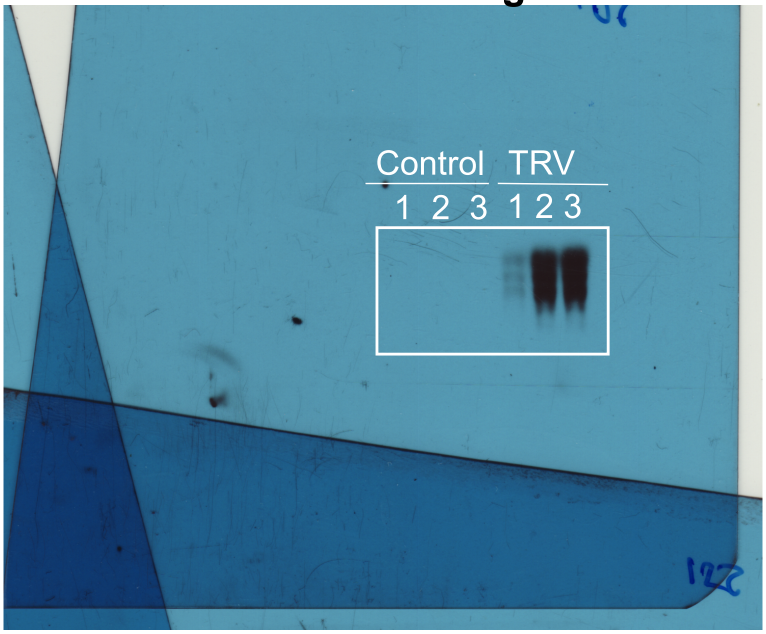

Ethidium bromide staining of rRNAs

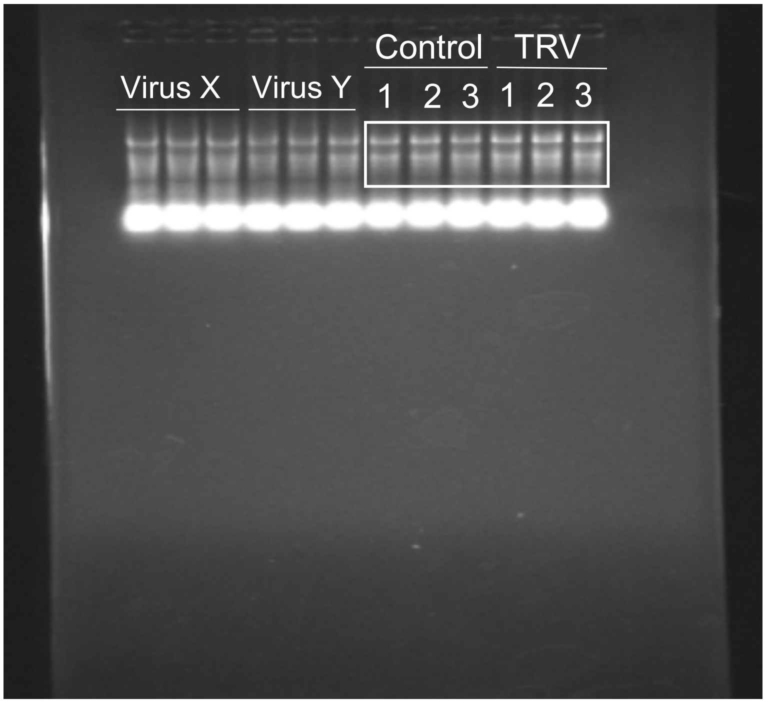

Note: The viruses X and Y samples in this gel were used for other projects.
